# Develop and Evaluate a New and Effective Approach for Predicting Dyslipidemia in Steel Workers

**DOI:** 10.3389/fbioe.2020.00839

**Published:** 2020-09-10

**Authors:** Jianhui Wu, Sheng Qin, Jie Wang, Jing Li, Han Wang, Huiyuan Li, Zhe Chen, Chao Li, Jiaojiao Wang, Juxiang Yuan

**Affiliations:** ^1^School of Public Health, North China University of Science and Technology, Tangshan, China; ^2^Hebei Province Key Laboratory of Occupational Health and Safety for Coal Industry, North China University of Science and Technology, Tangshan, China

**Keywords:** deep learning, convolutional neural network, dyslipidemia, steel worker, disease model prediction, model performance comparison

## Abstract

The convolutional neural network (CNN) has made certain progress in image processing, language processing, medical information processing and other aspects, and there are few relevant researches on its application in disease risk prediction. Dyslipidemia is a major and modifiable risk factor for cardiovascular disease, early detection of dyslipidemia and early intervention can effectively reduce the occurrence of cardiovascular diseases. Risk prediction model can effectively identify high-risk groups and is widely used in public health and clinical medicine. Steel workers are a special occupational group. Their particular occupational hazards, such as high temperatures, noise and shift work, make them more susceptible to disease than the general population, which makes the risk prediction model for the general population no longer applicable to steel workers. Therefore, it is necessary to establish a new model dedicated to the prediction of dyslipidemia of steel workers. In this study, the physical examination information of thousands of steel workers was collected, and the risk factors of dyslipidemia in steel workers were screened out. Then, based on the data characteristics, the corresponding parameters were set for the convolutional neural network model, and the risk of dyslipidemia in steel workers was predicted by using convolutional neural network. Finally, the predictive performance of the convolutional neural network model is compared with the existing predictive models of dyslipidemia, logistics regression model and BP neural network model. The results show that the convolutional neural network has a good predictive performance in the risk prediction of dyslipidemia of steel workers, and is superior to the Logistic regression model and BP neural network model.

## Introduction

Dyslipidemia is a chronic noncommunicable disease of lipid metabolism disorder, characterized by increased and/or decreased lipid levels in the blood. With the rapid development of China’s economy and the change of life style, cardiovascular disease has become the main death disease of residents (Roth et al., 2017). In recent years, the blood lipid level of Chinese population has gradually increased, and the prevalence of dyslipidemia has increased significantly. Evidence demonstrates that dyslipidemia is an independent and modifiable major risk factor for cardiovascular disease, and its level can significantly increase the incidence and mortality of cardiovascular disease (Pikula et al., 2015; Lee et al., 2017). Studies have shown (Miller, 2009; Hendrani et al., 2016; Stevens et al., 2016) that the early detection and management of high-risk groups with dyslipidemia can effectively reduce the incidence of cardiovascular disease, which can reduce the burden of cardiovascular disease and brings great social value.

China has a huge number of steel workers. Steel workers are a special occupational group, whose occupational environment is special, such as high temperature, noise, shift system and other special occupational exposure can cause or affect the occurrence of chronic diseases (Chauhan et al., 2014; Hedén Stahl et al., 2014; Tong et al., 2017; Wu et al., 2019b). Therefore, the prediction model of dyslipidemia in the general population is not suitable for steel workers. In order to improve the quality of life and health status of steel workers, it is urgent to establish a new risk prediction model of dyslipidemia in steel workers.

Logistics regression is a traditional prediction model, which is widely used in the field of disease prediction due to its clear parameter significance and easy to understand outcome indicators (Liu et al., 2018). However, its applicable conditions are relatively strict, which often limits the accuracy of its predictions. BP neural network is a widely used artificial neural network for disease prediction (Yao et al., 2019). Its good nonlinear processing ability and flexible grid structure make it have a good self-learning ability. However, it has a slow learning speed and is liable to fall into local minima, which makes its network promotion ability limited. Convolutional neural network is a kind of feedforward neural network with deep structure and convolution computation. The convolution structure can reduce the memory occupied by the neural network and has strong adaptability. It is good at mining local features of data and extracting global training features and classification, which has some advantages that traditional technologies do not have. In addition, the three key operations of convolution kernel, “local receptive field,” weight sharing and pooling, can effectively reduce the number of network parameters, significantly reduce the computational complexity, and alleviate the problem of model overfitting.

Based on thousands of physical examination data of steel workers, we established a convolutional neural network model to predict the risk of dyslipidemia of steel workers, and compared the prediction performance with the existing dyslipidemia prediction model. Overall, our study consists of three contributions:

1. Based on thousands of physical examination data of steel workers, we screened out the risk factors of dyslipidemia of steel workers, which can provide a basis for formulating early prevention strategies for dyslipidemia of steel workers.
2. Combine the characteristics of the data to set the corresponding parameters of the model, and use the convolutional neural network to predict the risk of dyslipidemia in steel workers. We found that the convolutional neural network has a good fit with the physical examination data of steel workers, and has a good prediction performance.
3. Compare the prediction performance of the convolutional neural network model with some of the existing dyslipidemia prediction models and find that the prediction performance of the convolutional neural network model is better. In this way, we can use convolutional neural networks to predict the risk of dyslipidemia of steel workers, so as to achieve the early prevention of dyslipidemia of steel workers and improve the health and quality of life of steel workers.

## Related Work

Disease risk prediction model is a very effective way for early detection of high-risk groups. In recent years, more and more studies on model prediction of dyslipidemia have been conducted, such as Xinghua Yang et al. (2018) established a logistics model of dyslipidemia using a longitudinal database based on Taiwanese MJ health checkups. Chongjian Wang et al. (2012) established an artificial neural network model to identify those at high risk of dyslipidemia in rural adult residents. Xiaoshuai Zhang et al. (2019) used a random forest survival model to predict the risk of dyslipidemia in Chinese Han adults. However, these studies are aimed at the general population, and there are few studies on the risk prediction of dyslipidemia in special occupational populations.

Convolutional neural network has been widely used in medical research and has shown good accuracy and generalization ability (Lee et al., 2018; Lin et al., 2018; Horiuchi et al., 2019; Wu et al., 2019a). However, no one has tried to establish and evaluate the effect of convolutional neural network model on predicting the risk of dyslipidemia in steel workers.

## Materials and Methods

### Study Population

This study was a cross-sectional survey. Based on the baseline data of the health effects cohort study of the occupational population in the Beijing-Tianjin-Hebei region, steel workers who had undergone occupational health examinations in a steel group company hospital from March 2017 to June 2017 were selected as the research objects. To be eligible, steel workers must on-the-job for at least 1 year, aged ≤60 years and free from incomplete health examination data. Ultimately, a total of 4655 steel workers were included in the study. All steel workers included in the study received written informed consent. According to the 2016 Chinese guidelines for the management of dyslipidemia in adults (Joint committee for guideline revision, 2018), the steel workers were divided into the dyslipidemia group and the non-dyslipidemia group. Dyslipidemia refers to the total cholesterol (TC) ≥ 6.2 mmol/L, and/or triglyceride (TG) ≥ 2.3 mmol/L, and/or low-density lipoprotein cholesterol (LDL-c) ≥ 4.1 mmol/L, and/or high-density lipoprotein cholesterol (HDL-c) < 1.0 mmol/L.

## Data Collection

The basic personal information of the steel workers was collected in a one-to-one questionnaire by trained investigators, which mainly included ethnicity, age, gender, education leve, marital status, income, family history of hyperlipidemia, drinking status, smoking status, physical activity, diet, etc. Anthropometric data are measured and collected by doctors and professional trainers in the physical examination center according to the unified standards, which mainly include weight, height, hip circumference, waist circumference, blood pressure, etc. Then the body mass index (BMI) is calculated by dividing the weight (kg) by the square of height (m), and the waist to hip ratio (WHR) is calculated by dividing the waist circumference (cm) by the hip circumference (cm). Laboratory test data were obtained by analyzing fasting blood samples of steel workers collected by doctors or nurses in the hospital, which mainly included total cholesterol (TC), triglycerides (TG), low-density lipoprotein cholesterol (LDL-C), high-density lipoprotein cholesterol (HDL-C), fasting blood glucose, etc. Occupational factors are provided by steel companies, which mainly include high temperature exposure, noise exposure, shift exposure, etc. Hypertension is defined as blood pressure ≥ 140/90 mmHg or diagnosed as hypertension by doctors. Diabetes is defined as FPG ≥ 7.0 mmol / L or diagnosed as diabetes by doctors.

### Model Independent Variable Filtering Method

We established an Excel database based on questionnaires and physical examination data, and screened out independent variables of risk factors for dyslipidemia of steel workers for model prediction. Measurement data was presented as $\bar{X}\pm S$ for normal distribution or M(P25, P75) for non-normal distribution, and we used *t*-test or the rank sum test for comparison between groups, respectively. The classification data was expressed by numbers and percentages, and the comparison between groups was performed by Chi-square test. The rank data was presented by numbers and composition ratio, and the rank sum test was used for inter group comparison. Unconditional Logistic regression analysis was used for multivariate analysis of influencing factors. Differences were deemed significant when *p* < 0.05. Factors influencing dyslipidemia of steel workers were screened out by univariate analysis and multi-factor logistics regression analysis. In order to avoid the influence of data multicollinearity, the screened influencing factors were diagnosed by multicollinearity. Combined with expert consultation and literature inquiry to determine the appropriate model independent variables. The statistical analysis was performed by SPSS 25.0. ROC curves were drawn using MedCalc.

### The Construction of Sample Set

**FIGURE 1:**
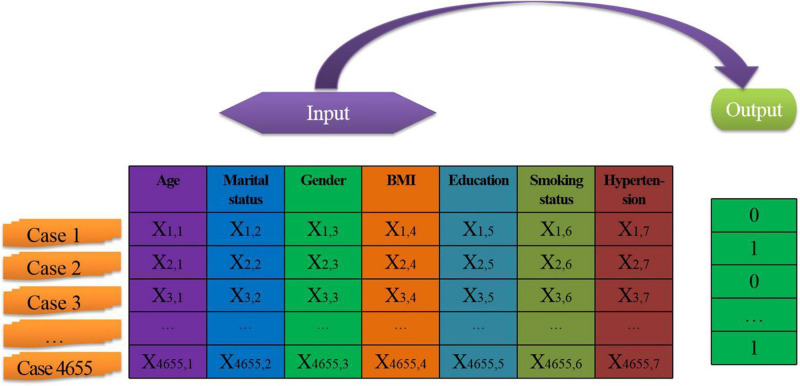
Sample data structure.

After screening (the screening results will be introduced later), a total of 4655 steel workers’ physical examination data constitute the sample set, as shown in Figure 1. There are seven independent variables, and the output target value is the presence or absence of dyslipidemia (Dyslipidemia is represented by 1 and non-dyslipidemia is represented by 0). Meanwhile, 4655 sample data were randomly assigned into 70% training set (*n* = 3258), 20% verification set (*n* = 931), and 10% test set (*n* = 466).

### Convolutional Neural Network Configuration

**FIGURE 2:**
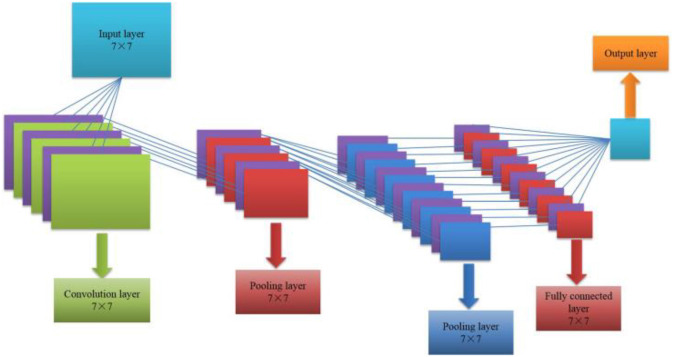
CNN algorithm structure.

Convolutional neural network is an important algorithm in the field of deep learning, including five parts of input layer, convolution layer, activation function, pooling layer and fully connected layer. It continuously adjusts the bias and connection weights between various neurons by combining forward propagation of information and backward propagation of error (Arun et al., 2018; Keshari et al., 2018). Its algorithm structure is shown in Figure 2.

**FIGURE 3:**
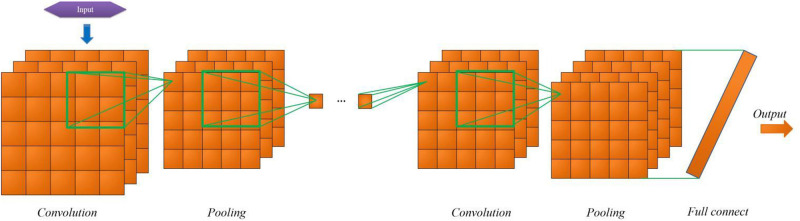
Structure of CNN prediction model for dyslipidemia in steel workers.

To predict whether steel workers are dyslipidemia by convolutional neural network, setting reasonable complex structure is an important premise to ensure the accuracy of the prediction model. According to the characteristics of the collected data, the network structure of convolutional neural network model designed is shown in Figure 3. We set up 1 input layer, 3 convolution layers, 3 pooling layers, 1 fully connected layer and 1 output layer in convolutional neural network. The size of the convolution kernel set by the convolution layer 1∼3 is 2 × 2, and the number of convolution nuclei is 20. All the three poolings are maximized sampling (Xu et al., 2015; Iqbal et al., 2018), the core size is 2 × 2. The activation functions are all Relu functions. The number of neurons in the whole connective layer is 25.

### Convolutional Neural Network Algorithm Solution

In this paper, we use the data of thousands of physical examination questionnaires of steel workers and convolution neural network algorithm to analyze and predict whether individuals have dyslipidemia.

A convolutional neural network for data processing rules that input data will pass through one or more hidden layers. In the hidden layer, each data is assigned a weight and bias, so the input data is assigned a new output value. If these new output values do not meet expectations, they are also assigned new weights and bias, and the process is repeated to produce the final output. The process mainly includes forward propagation and backward propagation.

The forward propagation calculation formula of convolutional neural network is:

(1) $$a_{j}^{\left(l\right)}=f\,\left(u^{l}\right)$$

(2) $$u^{l}=W^{l}\,a^{\left(l-1\right)}+b^{l}$$

Where $a_{j}^{\left(l\right)}$ represents the output of layer *l*, *W*^*l*^ represents weights, *b*^*l*^ represents biases, *f* is the activation function.

Since the feature map input in the forward propagation of the convolutional layer is convolved with the convolution kernel, the forward propagation formula of the *j*-th convolution kernel in the *l*-th layer of the convolutional neural network is as follows:

(3) $$a_{j}^{\left(l\right)}=f\,\left(\sum_{i\in N_{j}}a_{j}^{\left(l-1\right)}\cdot k_{i\,j}^{\left(l\right)}+b_{j}^{\left(l\right)}\right)$$

Where *k* is the convolution kernel, $a_{j}^{\left(l-1\right)}$ is the output of the *j*-th convolution kernel of the *l*-1 layer, *N*_*j*_ is a choice of input features. $b_{j}^{\left(l\right)}$ is the bias Shared by each convolution layer, *f* is the activation function of the convolution layer.

The forward propagation of the pooling layer requires the pooling calculation of the input features and then other calculations. The calculation formula of the pooling layer is as follows:

(4) $$a_{j}^{\left(l\right)}=f\,\left(\beta_{j}^{\left(l\right)}\,p\,o\,o\,l\,i\,n\,g\,\left(a_{j}^{\left(l-1\right)}\right)+b_{j}^{\left(l\right)}\right)$$

Where $a_{j}^{\left(l\right)}$ is the result of pooling the *j*-th characteristic map of the *l*-th convolution. *Pooling* is the pooling operation, $\beta_{j}^{\left(l\right)}$ is the multiplicative bias of the pooling layer, and $b_{j}^{\left(l\right)}$ is the additive bias of the pooling layer.

The back propagation of convolutional neural network. Suppose that the loss function *J*_*mse*_ defined as the convolution neural network is the mean square error, and the formula is as follows:

(5) $$J_{m\,s\,e}=\frac{1}{2}\,\sum_{i=1}{\left(Y_{i}-y_{i}\right)}^{2}$$

Where *Y*_*i*_ is the actual value and *y*_*i*_ is the output value.

Backpropagation of the fully connected layer in convolutional neural network is obtained by BP algorithm. For the convolution layer of the convolution neural network, if the next layer of the convolution layer *l* is the fully connected layer, then the sensitivity $\delta_{j}^{\left(l\right)}$ of the *j*-th convolution kernel can be obtained by the BP algorithm. If it is the pooling layer, then the calculation formula of the error sensitivity is:

(6) $$\delta_{j}^{\left(l\right)}=\beta_{j}^{\left(l+1\right)}\,\left(f^{\prime }\,\left(u_{j}^{\left(l\right)}\right)\circ u\,p\,\left(\delta_{j}^{\left(l+1\right)}\right)\right)$$

Where $\beta_{j}^{\left(l+1\right)}$ represents the multiplier bias of the corresponding pooling layer, and *up* represents the anti-pooling operation. After the error sensitivity of the convolution layer is obtained, the convolution kernel and bias of the convolution layer are updated, and the formula is as follows:

(7) $$\frac{\partial J_{m\,s\,e}}{\partial k_{i\,j}^{\left(l\right)}}=\sum_{m,n}\delta_{j}^{\left(l\right)}\,p_{j}^{\left(l-1\right)}$$

(8) $$\frac{\partial J_{m\,s\,e}}{\partial b_{j}}=\sum_{m,n}{\left(\delta_{j}^{\left(l\right)}\right)}_{m,n}$$

Where $p_{j}^{\left(l-1\right)}$ is the value of $a_{j}^{\left(l-1\right)}$ multiplied by each element of the convolution kernel $k_{i\,j}^{\left(l\right)}$, *m* and *n* are the location information of the element in the input feature.

Similarly, the pooling layer is similar to the convolution layer. When the pooling layer is followed by the fully connected layer, the error sensitivity can be obtained by BP algorithm. When the pooling layer is the convolution layer, the error sensitivity is:

(9) $$\delta_{j}^{\left(l\right)}=f^{\prime }\left(u_{j}^{\left(l\right)}\right)\circ conv2\left(\delta_{j}^{\left(l+1\right)},rot180\left(k_{j}^{l+1}\right){,}^{\prime }full^{\prime }\right)$$

Where *conv2* represents the convolution calculation, *rot180* represents the rotation of the matrix by 180 degrees, and *full* represents the missing data in the matrix replaced by 0. After the error sensitivity of the pooling layer is obtained, the gradient calculation formula of $b_{j}^{\left(l\right)}$ and $\beta_{j}^{\left(l\right)}$ is as follows:

(10) $$\frac{\partial J_{m\,s\,e}}{\partial b_{j}}=\sum_{m,n}{\left(\delta_{j}^{\left(l\right)}\right)}_{m,n}$$

(11) $$\frac{\partial J_{m\,s\,e}}{\partial \beta_{j}}=\sum_{m,n}{\left(\delta_{j}^{\left(l\right)}\circ p\,o\,o\,l\,i\,n\,g\,\left(a_{j}^{\left(l-1\right)}\right)\right)}_{m,n}$$

### Platform and Parameter Settings

In this paper, TensorFlow modules in Python are used to construct the convolutional neural network model. TensorFlow is fully open source and available to anyone with minimal device configuration requirements. It can run models automatically on all platforms, from mobile phones, a single CPU/GPU, to distributed systems consisting of hundreds of GPU CARDS.

**FIGURE 4:**
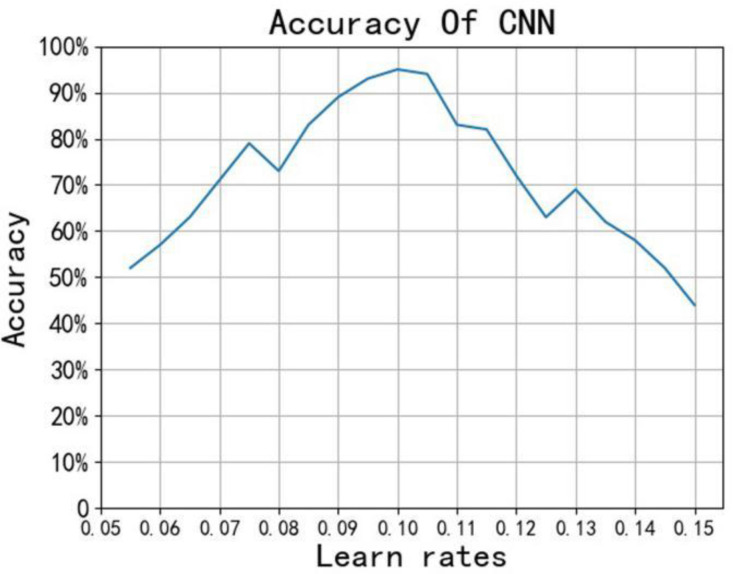
Learning rate and accuracy.

We use the random initialization function to set the weight and bias. The smaller the learning rate, the longer the model takes to converge, but it can improve the accuracy of the model. In order to find the best learning rate of convolutional neural network, we first use random function in Python to initialize the learning rate at random, and then use Python to traverse different learning rates in steps of 0.01. Finally, use Matplotlib module to make some corresponding images as shown in Figure 4. It can be seen that when the learning rate is about 0.1, the accuracy is the highest. Therefore, choosing 0.1 as the learning rate can make the convolutional neural network achieve better prediction effect.

### Performance Metrics

In this paper, five performance metrics including accuracy, sensitivity, specificity, F1-score and ROC curve were selected to evaluate the performance of the convolutional neural network model. Meanwhile, the prediction performance of training set and test set of convolutional neural network model, Logistics regression model and BP neural network model was compared. The calculation method of the above metrics are as follows:

(12) $$\text{Sensitivity}=\frac{\text{TP}}{\text{TP}+\text{FN}}$$

(13) $$\text{Specificity}=\frac{\text{TN}}{\text{TN}+\text{FP}}$$

(14) $$\text{Accuracy}=\frac{\text{TP}+\text{TN}}{\text{TP}+\text{FP}+\text{TN}+\text{FN}}$$

(15) $$F_{1}=2\cdot \frac{p\,r\,e\,c\,i\,s\,i\,o\,n\cdot r\,e\,c\,a\,l\,l}{p\,r\,e\,c\,i\,s\,i\,o\,n+r\,e\,c\,a\,l\,l}$$

$$p\,r\,e\,c\,i\,s\,i\,o\,n=\frac{\text{TP}}{\text{TP}+\text{FP}}$$

$$r\,e\,c\,a\,l\,l=\frac{\text{TP}}{\text{TP}+\text{FN}}$$

TP represents true positions, TN represents true negatives, FP represents false positions, FN represents false negatives. Sensitivity reflects the model’s ability to find patients, specificity reflects the model’s ability to find non-patients, and accuracy represents the model’s overall predictive ability. The F1 score is a harmonic average of the accuracy and recall rates and is used as a final measurement. In addition, ROC curves and AUC are often used to test the balance between true and false positive rates.

## Results

### Baseline Characteristics

A total of 4655 subjects were included in this study, including 1795 cases of dyslipidemia (38.56%) and 2860 cases of non-dyslipidemia (61.43%). The characteristics of baseline data and the results of univariate analysis are shown in Table 1. Univariate analysis showed that there were statistically significant differences (*p* < 0.05) between the dyslipidemia group and the non-dyslipidemia group in gender, educational level, marital status, smoking status, drinking situation, hypertension, diabetes, BMI, waist-to-hip ratio, shift work, and occupational high temperature. Unexpectedly, no significant differences (*p* > 0.05) were observed in ethnicity, age, income, diet, physical activity, family history of hyperlipidemia and occupation noise between the two groups.

**TABLE 1:** Comparison of baseline characteristics of dyslipidemia and non-dyslipidemia patients in steel workers.

Variable	Dyslipidemia *N* (%)/*M* (P25,P75)		*χ*^2^/Z	*p*
	No (*N* = 2860)	Yes (*N* = 1795)		
Age	46 (38,50)	46 (39,50)	0.739	0.46
Gender			54.494	<0.001
Male	2549 (89.1)	1711 (95.3)		
Female	311 (10.9)	84 (4.7)		
Nation			1.834	0.176
Han	2801 (97.9)	1747 (97.3)		
Other	59 (2.1)	48 (2.7)		
Marital status			2.0046	<0.001
Unmarried	119 (4.2)	34 (1.9)		
Married	2670 (93.4)	1702 (94.8)		
Other	71 (2.5)	59 (3.3)		
Education			18.67	<0.001
Elementary and below	37 (1.3)	18 (1.0)		
Middle and high school	2142 (74.9)	1408 (79.4)		
Junior college and undergraduate	639 (22.3)	363 (20.2)		
Graduate and above	42 (1.5)	6 (0.3)		
Monthly income	6000 (4000,7000)	5000 (4000,7000)	0.259	0.796
Family history of hyperlipidemia			0.976	0.323
No	2724 (95.2)	1698 (94.6)		
Yes	136 (4.8)	97 (5.4)		
Smoking status			93.918	<0.001
No smoking	1374 (48.0)	605 (33.7)		
Quit smoking	149 (5.2)	105 (5.8)		
smoking	1337 (46.7)	1085 (60.4)		
Drinking situation			11.509	0.003
No drinking	1762 (61.6)	1016 (56.6)		
Quit drinking	58 (2.0)	40 (2.2)		
Drinking	1040 (36.4)	739 (41.2)		
Physical activity			0.247	0.884
Mild	616 (21.5)	378 (21.1)		
Moderate	1233 (43.1)	786 (43.8)		
Severe	1011 (35.3)	631 (35.2)		
High fat diet score	12 (11,13)	12 (11,13)	0.916	0.36
Vegetable Fruit Score	6 (6,7)	6 (5,7)	1.748	0.08
BMI	24.9 (22.7,27.2)	26.7 (24.5,29.1)	17.277	<0.001
WHR	0.875 (0.831,0.917)	0.901 (0.862,0.939)	13.4	<0.001
Diabetes			10.448	0.001
No	2751 (96.2)	1690 (94.2)		
Yes	109 (3.8)	105 (5.8)		
Hypertension			34.381	<0.001
No	2480 (86.7)	1441 (80.3)		
Yes	380 (13.3)	354 (197)		
Shift work			6.276	0.043
Never shift	500 (17.5)	269 (15.0)		
Once shifts	509 (17.8)	306 (17.0)		
Now shifts	1851 (64.7)	1220 (68.0)		
Occupation noise			3.02	0.082
No	2279 (79.7)	1392 (77.5)		
Yes	581 (20.3)	403 (22.5)		
Occupation high temperature			7.288	0.007
No	2373 (83.0)	1433 (79.8)		
Yes	487 (17.0)	362 (20.2)		

### Independent Variable Selection

The significant variables of univariate analysis were used for multicollinearity diagnosis, and age as an influential factor of disease was also included in the analysis. The results show (Table 2) Tolerance > 0.1 and VIF < 10, so there is no multicollinearity among the variables. Then, these variables were analyzed by multivariate unconditional logistic regression. The results showed (Table 3) that marital status and educational level were the influencing factors of dyslipidemia. Meanwhile, hypertension is a risk factor for dyslipidemia, male workers have lower risk than female workers, the steel workers who don’t smoke have a lower risk. The higher the BMI, the higher the risk of dyslipidemia. Literature supports (Ni et al., 2015; Pereira et al., 2015; Qi et al., 2015) that age is an influential factor of dyslipidemia, so age was included in the model as an independent variable. Finally, according to factor analysis, literature inquiry and expert consultation, seven independent variables were selected to enter the model. The seven independent variables are age, gender, marital status, educational, BMI, smoking status and hypertension.

**TABLE 2:** Multicollinearity diagnostic table.

	Collinearity statistics	
	Tolerance	VIF
(Constant)		
Age	0.711	1.407
Marital status	0.91	1.099
Gender	0.873	1.146
BMI	0.936	1.069
WHR	0.979	1.021
Education	0.758	1.319
Smoking status	0.849	1.178
Drinking situation	0.88	1.136
Diabetes	0.966	1.035
Hypertension	0.91	1.098
Shift work	0.966	1.035
Occupation high temperature	0.991	1.009

**TABLE 3:** Multivariate logistics regression analysis of risk factors of dyslipidemia in steel workers.

Variable	B	S.E.	Wald	df	Sig.	Exp (B)	95% C.I. for Exp (B)	
							Lower	Upper
Marital status (others)								
Unmarried	–0.963	0.287	11.285	1	0.001	0.382	0.218	0.67
Gender (female)	–0.479	0.137	12.193	1	0	0.619	0.473	0.81
BMI	0.13	0.009	198.068	1	0	1.139	1.118	1.159
Education (graduate and above)								
Middle and high school	1.072	0.452	5.612	1	0.018	2.921	1.203	7.091
Junior college and undergraduate	1.035	0.452	5.253	1	0.022	2.815	1.162	6.821
Smoking status (smoking)			44.924	2	0			
No smoking	–0.473	0.071	44.821	1	0	0.623	0.542	0.716
Hypertension	0.187	0.088	4.521	1	0.033	1.206	1.015	1.434

### Convolutional Neural Network Model Results

**FIGURE 5:**
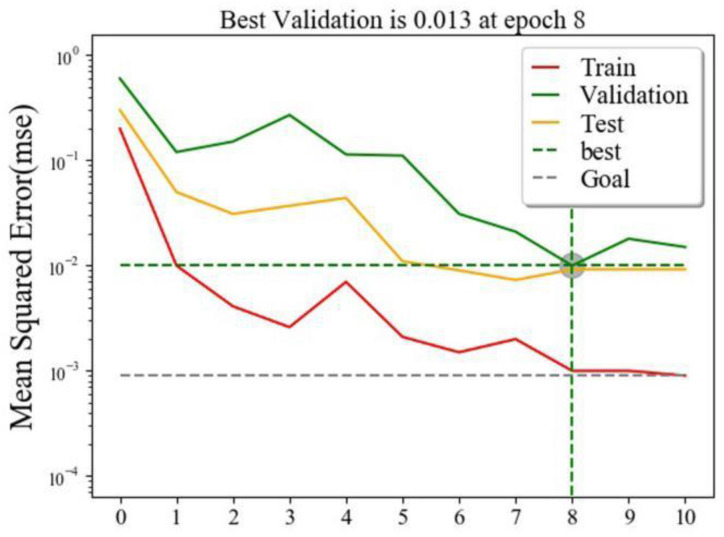
Effect error graph of CNN learning.

**FIGURE 6:**
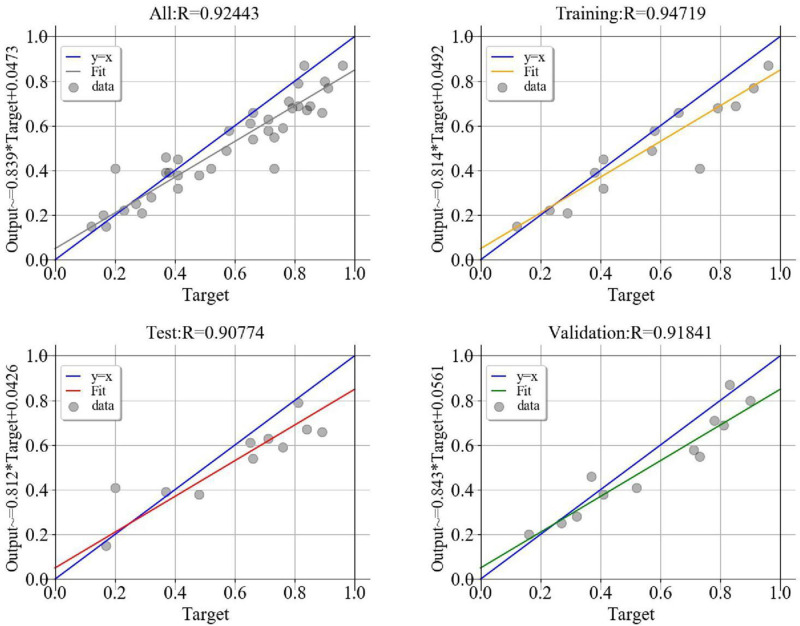
CNN model goodness of fit test chart.

The effect error chart (Figure 5) of the dyslipidemia convolutional neural network prediction model shows that the minimum verification error is 0.013 when training in step 8. The goodness of fit test results of the convolutional neural network prediction model for dyslipidemia (Figure 6) show that the training set is 0.974, the verification set is 0.918, and the test set is 0.908. The performance metrics of the convolutional neural network model of dyslipidemia in steel workers are shown in Table 4. The sensitivity is 93.23, 90.00, and 89.97% in training set, test set and verification set, respectively. The specificity is 95.65, 91.26, and 93.01% in training set, test set and verification set, respectively. The accuracy is 94.72, 90.77, and 91.84% in training set, test set and verification set, respectively. The F1 score is 0.93, 0.91, and 0.89 in training set, test set and verification set, respectively. The AUC (95% CI) is 0.944 (0.936–0.952), 0.906 (0.876–0.931) and 0.915 (0.895–0.932) in training set, test set and verification set, respectively. The above results show that the convolutional neural network model is very suitable for the physical examination data of steel workers with dyslipidemia. In addition, the convolutional neural network model has a good ability to find patients with dyslipidemia and non-dyslipidemia, and has high prediction accuracy.

**TABLE 4:** Comparison of performance metrics of each model.

Model	Training set				Test set			
	Sensitivity (%)	Specificity (%)	Accuracy (%)	F1 score	Sensitivity (%)	Specificity (%)	Accuracy (%)	F1 score
Logistics	72.45	76.47	74.92	0.69	71.11	70.30	70.6	0.65
BP neural network	86.7	88.96	88.09	0.85	81.11	83.57	82.62	0.78
CNN	93.23	95.65	94.72	0.93	90.00	91.26	90.77	0.88

### Model Effect Comparison

We compared the prediction performance of the convolutional neural network model for dyslipidemia in steel workers with that of the Logistics regression model and BP neural network model. The comparison results of performance metrics are shown in Table 5.

**TABLE 5:** Performance metrics of convolutional neural network.

Performance metrics	Training set	Test set	Validation set
Sensitivity (%)	93.23	90.00	89.97
Specificity (%)	95.65	91.26	93.01
Accuracy (%)	94.72	90.77	91.84
F1 score	0.93	0.91	0.89
AUC (95% CI)	0.944 (0.936–0.952)	0.906 (0.876–0.931)	0.915 (0.895–0.932)

**FIGURE 7:**
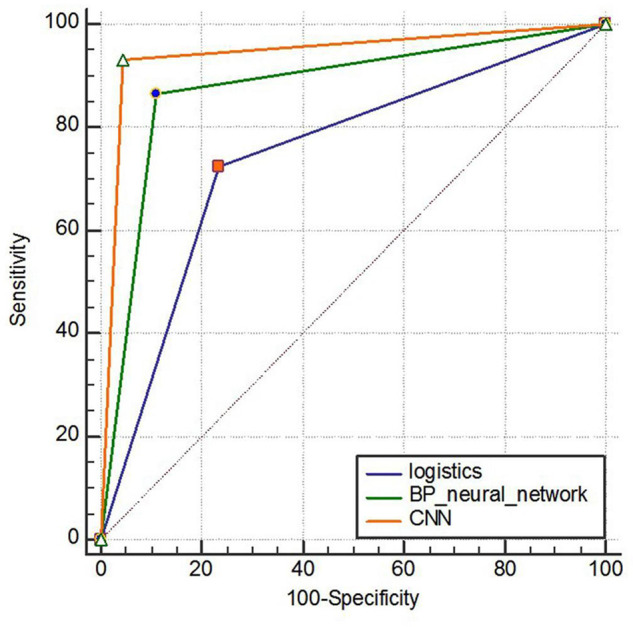
ROC curve comparison of three model training sets.

Predictive performance results of three model training sets samples. The sensitivity of the logistic regression model, BP neural network model and convolutional neural network model is 72.45%, 86.7% and 92.23%, respectively. The specificity is 76.47, 88.96, and 95.65%, respectively. The accuracy is 74.92, 88.09, and 94.72%, respectively. The F1 score is 0.69, 0.85, and 0.93, respectively. The area under the ROC curve is shown in Figure 7, and the AUC (95%CI) is 0.745 (0.729–0.760), 0.878 (0.867–0.889), and 0.944 (0.936–0.952), respectively, with statistically significant differences (*P* < 0.001).

**FIGURE 8:**
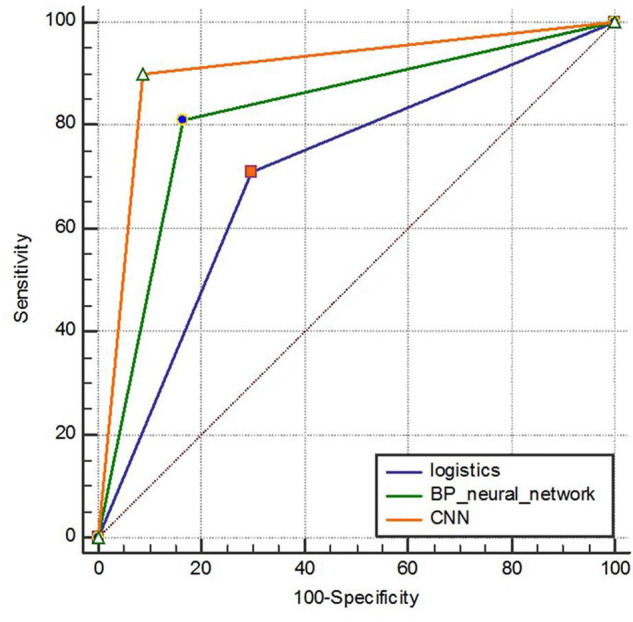
ROC curve comparison of three model test sets.

Predictive performance results of three model test sets samples. The sensitivity of the logistic regression model, BP neural network model and convolutional neural network model is 71.11, 81.11, and 90.00%, respectively. The specificity is 70.30, 83.57, and 91.26%, respectively. The accuracy is 70.60, 82.62, and 90.77%, respectively. The F1 score is 0.65,0.78 and 0.88, respectively. The area under the ROC curve is shown in Figure 8, and the AUC (95%CI) is 0.707 (0.663–0.748), 0.823 (0.786–0.857), and 0.906 (0.876–0.931), respectively, with statistically significant differences (*P* < 0.001).

In combination with the above performance metrics, in the prediction of dyslipidemia of steel workers, the convolutional neural network is optimal in terms of sensitivity, specificity, accuracy, F1 score and AUC. Therefore, in the prediction of dyslipidemia in steel workers, the convolutional neural network has better prediction performance.

## Conclusion

In this work, we constructed a convolutional neural network model to predict dyslipidemia in steel workers, a special occupational group. At the beginning, we screened the data and found out the risk factors for dyslipidemia in steel workers to construct a prediction model. Subsequently, we tested the fitting degree of the model and data, and the goodness of fit in the training set, test set and verification set were 94.72, 90.77, and 91.84%, respectively. In addition, we evaluate the prediction performance of the convolution neural network model. In the training set, test set and verification set, the sensitivity is 93.23, 90.00, and 89.97%, respectively. The specificity is 95.65, 91.26, and 93.01%, respectively. The accuracy is 94.72%, 90.77% and 91.84%, respectively. The F1 score is 0.93, 0.91, and 0.89, respectively. The AUC (95% CI) is 0.944 (0.936–0.952), 0.906 (0.876–0.931) and 0.915 (0.895–0.932), respectively. The results prove that the convolutional neural network is very suitable for the prediction of dyslipidemia of steel workers and has high accuracy.

Finally, we compared the predictive performance of the convolutional neural network with the logistics model and BP neural network model of common models of dyslipidemia. We found that the predictive performance of the convolutional neural network model was better than that of the Logistics regression model and BP neural network model in the risk prediction of dyslipidemia of steel workers.

In the current study, the convolutional neural network model can accurately predict the risk of dyslipidemia in steel workers, and is superior to some existing predictive models of dyslipidemia. Therefore, the convolutional neural network model can be used to predict the risk of dyslipidemia in steel workers, and provide a basis for the formulation of early prevention strategies for dyslipidemia in steel workers, so as to improve the health status and quality of life of steel workers. In this paper, we only use the traditional convolutional neural network algorithm. So in the future, we will further study new algorithms to improve the predictive performance of the model.

## Data Availability Statement

The datasets presented in this article are not readily available because of moral restrictions. Requests to access the datasets should be directed to the corresponding author.

## Ethics Statement

This research was approved by the Ethics Committee of North China University of Science and Technology. The participants provided their written informed consent to participate in this study.

## Author Contributions

HW and SQ contributed to conception and design of the study. JW and JL organized the database. HW and HL performed the statistical analysis. SQ wrote the first draft of the manuscript. ZC, CL, and JJW wrote sections of the manuscript. JY contributed to manuscript revision. All authors agreed to submit this article.

## Conflict of Interest

The authors declare that the research was conducted in the absence of any commercial or financial relationships that could be construed as a potential conflict of interest.
